# Obituary

**Published:** 1869-04

**Authors:** W. C. Horne


### OBITUARY.

DIED, at his residence, No. 18 West 11th Street, New York City, Friday evening, January 8th, Alfred W. Allen, in the forty-third year of his age.

"At a meeting of Dentists, held at Mott Library, January 13, 1869, the occasion being the death of Dr. A. W. Allen; Dr. J. G. Ambler was called to the chair, and Dr. W. C. Horne was appointed Secretary. Appropriate remarks were made by Drs. Ambler, Northrop, and Atkinson, and on motion of Dr. Horne, a committee of three was appointed to prepare suitable resolutions expressive of the sympathy of the meeting with the family of the deceased, and to publish them in such journals as they should deem proper. The committee appointed by the meeting consists of Drs. Ambler, Northrop, and Horne."

### RESOLUTIONS.

WHEREAS, We have heard with deep regret of the sudden death of Dr. A. W. Allen, Dentist, of this city, taken away in the midst of a life of usefulness. Therefore,

*Resolved*, That we bear testimony to the high regard in which we held the deceased, as a professional man, and as a Christian gentleman; one who in all the walks of life was actuated by the desire to be good and to do good, and who in his honorable calling earned the esteem of his patients, and of us, his fellows, for his ability and faithfulness.

*Resolved*, That we tender to the widow of our departed friend, his sister, and to his beloved brother, Dr. Wm. H. Allen, the assurance of our sorrowful sympathy in their great affliction.

W. C. HORNE, *Secretary.*

